# Assessment of the opportunities for increasing the availability of EU data on consumer product-related injuries

**DOI:** 10.1136/injuryprev-2020-043677

**Published:** 2020-05-05

**Authors:** Anita Radovnikovic, Otmar Geiss, Stylianos Kephalopoulos, Vittorio Reina, Josefa Barrero, Silvia Dalla Costa, Marco Verile, Eleonora Mantica

**Affiliations:** 1 European Commission, Joint Research Centre (JRC), Ispra, Italy; 2 European Environment Agency (EEA), Kobenhavn, Denmark

**Keywords:** policy, policy analysis, qualitative research, registry, surveillance

## Abstract

The availability of data on consumer products-related accidents and injuries is of interest to a wide range of stakeholders, such as consumer product safety and injury prevention policymakers, market surveillance authorities, consumer organisations, standardisation organisations, manufacturers and the public. While the amount of information available and potentially of use for product safety is considerable in some European Union (EU) countries, its usability at EU level is difficult due to high fragmentation of the data sources, the diversity of data collection methods and increasing data protection concerns. To satisfy the policy need for more timely information on consumer product-related incidents, apart from injury data that have been historically collected by the public health sector, a number of 'alternative' data sources were assessed as potential sources of interest. This study explores the opportunities for enhancing the availability of data of consumer product-related injuries, arising from selected existing and 'alternative' data sources, widely present in Europe, such as firefighters’ and poison centres’ records, mortality statistics, consumer complaints, insurance companies’ registers, manufacturers’ incident registers and online news sources. These data sources, coupled with the use of IT technologies, such as interlinking by remote data access, could fill in the existing information gap. Strengths and weaknesses of selected data sources, with a view to support a common data platform, are evaluated and presented. Conducting the study relied on the literature review, extensive use of the surveys, interviews, workshops with experts and online data-mining pilot study.

## Introduction

Safety of consumer products is a prerequisite for placing products on the European Union (EU) market.^1^ This requirement is ensured by a complex framework of regulations covering general product safety and sector/product specific legislation (eg, for electrical and electronic goods and toys) which are supported by product-specific standards.^1–3^

Despite the solid product safety regulatory framework in the EU, estimates from available publications indicate that around three-quarters of all unintentional injuries are home and leisure injuries.^4 5^ A proportion of these injuries may be related to the improper use of or an underlying safety issue with consumer products, but at present it appears challenging to accurately quantify the extent of product-related injuries and accidents at the EU level.

Information on circumstances of accidents leading to injuries can help revealing unsafe practices in handling consumer products, the mechanism of injury, new products posing risks or in the definition of appropriate safety requirements by standardisation and safety by design. Data collection methodologies used for injury surveillance and injury prevention strategies have existed for a long time and are well documented.^6^

In Europe, based on Council Recommendation (2007/C 164/01) on the prevention of injury and the promotion of safety, national authorities are required to collect data on injuries and on those ‘caused by products and services’. In the Regulation (EC) No 765/2008 setting out the requirements for accreditation and market surveillance related to the marketing of products, Article 18 defines that Member States are required to ‘establish adequate procedures in order to […] monitor accidents and harm to health which are suspected to have been caused by those products; […]’.^7^

Regulation (EC) No 1338/2008 defines the provision of data on healthcare, causes of death (COD), accidents at work and occupational disease. Annex I of this Regulation lists necessary data sets such as ‘accidents and injuries, including those related to consumer safety’ that are required to be compiled by national authorities through the use of the European Health Interview Survey (EHIS) (or other administrative sources) and transmitted to Eurostat at least every 5 years.^8^ As evident from the last EHIS questionnaire, the level of detail on external causes of injuries collected by EHIS is insufficient on consumer product-related injuries.

Traditionally, data collection on injuries is dealt with by national systems for all injury (intentional and unintentional) surveillance which systematically collect data from emergency departments or hospital discharge registers. Classification of external causes of injuries may be handled by in-house or existing codifications systems with a multidimensional approach like Nordic Medico-Statistical Committee (NOMESCO) classification or International Classification of External Causes of Injuries (ICECI), or those used for injuries, burns and poisonings, within International Classification of Diseases (ICD)-9 and ICD-10 (ICD-11 is under preparation).^9 10^ The state of EU injury surveillance, available data sources, their weaknesses and the stakeholders who can benefit from ‘all injury’ data collection systems have already been critically assessed by other authors.^11–15^ While the amount of information available for injury prevention and product safety policy work at national level is considerable in some EU countries, its usability at EU level is difficult due to high regional fragmentation of the data sources, different methodologies and increasing data privacy issues (especially those concerning access to hospital data). Injury surveillance and injury information generated in the EU have been previously described as ‘operating on an incomplete puzzle of data sources’.^11^

The need for better injury data, in particular the need for a sustainable pan-European accident and injury database, has already been proposed in a joint call by a large group of stakeholders, including business and consumer organisations such as the European consumer voice in standardisation (ANEC) and the European Consumer Organisation (BEUC).^16^ Such a database would be similar to the current most effective system for monitoring consumer product-related injuries, the US’s National Electronic Injury Surveillance System (NEISS), which is in operation since the 1970s.

On the international scene, the OECD’s Working Party on Consumer Product Safety has also recognised the importance of improving injury data availability as a tool for protecting consumers and proposed the establishment of a platform for global pooling of injury data (Global Injury Data portal).^17^ However, this initiative has not been carried out yet.

Globalised and quickly changing market of consumer products pose a challenge for jurisdictions involved in managing consumer safety. There is an increasing need for real-time data and a demand for quick action in addressing emerging public safety issues. In particular this has been recognised in the area of injury prevention and epidemiology, where these needs have driven groups of international injury prevention experts to promote a shift from passive to active surveillance by harnessing new information technologies, such as data linkage and moving from single source to multisource-based surveillance.^18^ To address the need for quick action, the strategic initiatives of the US Consumer Product Safety Commission (US CPSC) for 2018–2022 mention the need for enhancing in-house capacity for data mining and harnessing of alternative data sources for hazard analyses or monitoring purposes.^19^

A number of databases that are serving non-hospital-based sectors may contain information that could be of use for filling the existing data need. For example, databases maintained by fire brigades and rescue services could deliver useful information on specific accidents that involve consumer products. Likewise, the information received by national poison centres (PC) could be useful to timely identify unsafe consumer products or unsafe ways of handling or packaging such products that cause harmful exposure. In addition, business operators who record customer complaints could also provide valuable data. A considerable amount of information on emerging risks can nowadays be gathered from consumer complaints and through online news sources. The later has been further explored during the project by the use of the tool Europe Media Monitor (EMM).

This study presents the analysis of the strengths and weaknesses of existing data registers from different sectors to support an information platform for integrating heterogeneous data by remote access. The use of such platform could provide useful information to a wide range of stakeholders, including policymakers.

To our knowledge, assessing the opportunities for the use of already existing and ‘alternative’ non-health-based information sources for injury prevention, coupled with articles retrieved from online data mining and the use of remote data access for interlinking these sources onto a common platform to fill the current data gap in consumer product-related injury data in Europe, is original and has not been published by other authors.

## Methodology

### Mapping of existing data sources

#### Systematic Literature Review

A systematic literature review was conducted based on a simplified version of the Preferred Reporting Items for Systematic Review and Meta-Analyses methodology.^20^ A list of search terms used for literature review is given in table 1. Over 1000 abstracts have been reviewed for relevance in the searched scientific databases (Science Direct, Scopus, PubMed). Google search was also carried out. This step provided the first list of existing databases/data sources on injuries and accidents of potential relevance for consumer product safety and it provided the names of the experts in the field of consumer product safety and injury prevention. The limitations of the literature review are summarised below.

**Table 1:** Search terms used for literature review

Search terms		
Accident database	Injury database	EU accident database
EU injury database	Consumer products safety information database	Consumer products safety information
Injury surveillance	Data collection systems on injuries and accidents	Product-related injuries
Accident monitoring system	Accident surveillance	Accident AND consumer products
Accident AND data collection system	Accident and market surveillance	Accident AND product safety
Injury AND consumer products	Injury AND data collection system	Injury AND product safety
Consumer products AND accident	Consumer products AND injury	Consumer products AND database
Consumer products AND injury surveillance	Consumer products AND surveillance	Database AND product safety
Morbidity AND product safety	Mortality AND product safety	Injury AND consumer product AND database
Injury AND product safety AND database	Consumer product AND injury AND database	Consumer product AND accident AND database
Accident AND product safety AND EU	Injury AND product safety AND EU	Injury database AND EU accident database AND EU

· Search period: 2000–2016.

· Search terms present in abstract and title.

· Focus on sources written in English language.

· Search through scientific journals only (not in books).

While these limitations need to be noted, none of them however are considered critical in terms of the overall conclusions of this study.

### Survey of institutions from different sectors of activity

To avoid the under-representation of the non-academic type of institutions, the scientific literature review was complemented with a questionnaire survey. A number of experts across the EU (around 80), from various institutions and sectors, took part in a survey (via email, phone) on existing injury data collection practices in their institutions, of potential relevance to consumer product safety. Some of the survey questions were:

- What level of detail is available on involved products? (category or more detailed information on the product/brand name/batch?).
- What level of detail is available on circumstances of accidents? (narrative? coding methodology?).
- Type of injury data collected (injury standardised codification system in place?).
- How is the system fed? (hospital/emergency departments, rescue service reports, police).

The profile of sectors of activity that were involved in the survey was: public healthcare sector (34%), the firefighters and rescue services (FRS) (13%), PC (17%), industry associations and manufacturers from toy industry (3%), insurance associations and insurance companies (12%), research institutes (13%), consumer associations (5%), independent consultants on consumer safety (3%).

#### Survey of Consumer Safety Network

The Consumer Safety Network (CSN) is an expert group made up of national experts from the administrations of EU countries, as well as Norway, Iceland and Liechtenstein, chaired by the European Commission, which decides on priority issues related to consumer product safety in the EU. The aim of the questionnaire sent to the CSN was to find out what is the current status of existing publicly run systems for collecting consumer complaints on unsafe products and injury events (as referred to in Article 9, Paragraph 2 of the General Products Safety Directive (GPSD)).^21^ Responses were collected from CNS in the period from 27 February to 30 March 2017. The questionnaire included inquiries about:

- In what way can an interested party submit complaints on consumer products to the competent authority?
- Details of your system (eg, description, web address)
- Is there a specific coding/categorisation in place for complaints on consumer product safety?
- How many dangerous products, injuries and near-miss events are reported annually by consumers through customer complaints?
- Are your data on consumer complaints publicly available?
- Would you make your register of consumer product safety complaints available for EU policy and research purposes?

### Europe Media Monitor

The monitoring and extracting of online news sources that contain some information on injuries/accidents caused by unsafe toys has been carried out by EMM in a pilot study. EMM is a publicly accessible family of applications, developed and launched in 2002, as a real-time multilingual news monitoring and analysis tool, whose functionalities have been described by other authors.^22–25^ EMM gathers an average of 300 000 online news articles per day in up to 70 languages. It is used for multilingual news analyses by EU institutions, Member State authorities, international organisations, such as the United Nations and the African Union. Four groups of 'triggering' keywords were created for the ‘Toy’ pilot category:

- ‘Product’ (eg, hoverboard%, inflatable +toy%, lego+block%, lego%, magnet, magnetic +toy% …).
- ‘Hazard’ (eg, poisoning +hazard%, poisoning, suffocate, suffocation, swallow%, strangulate% …)
- ‘Consequences’ (eg, serious +injr%, hospital%, safety +risk%, death, died …)
- ‘Subjects’ (eg, boy, child, children, infants, …) followed by the ‘Exclusion keywords’ group which contained eliminatory words (eg, road accident, homicide, war) with the aim to avoid ‘collecting’ irrelevant articles.

EMM can ‘browse’ online open sources (news, social media, blogs) every 5 min for 24 hours each day. A list of 2593 online news providers in the English language has been used in this study.^26^ The list can be customised according to the domain-specific media monitoring needs.

### Workshops

During the project two experts workshops were organised with the aim to discuss the issue of increasing the availability of consumer product-related injury data with leading European experts in injury prevention, consumer product safety, mortality statistics, EU policy officers, independent consultants, safety researchers, representatives of firefighters associations and PC. The first workshop was carried out soon after the literature review and the survey on available data on injuries from different sectors in the EU have been conducted (October 2016). A second workshop has been carried out at the end of the study where the outcomes of the study were presented and discussed (November 2017).

### SWOT analyses

A potential of selected data sources to provide long-term support to integrated IT information platform on consumer product-related injuries and accidents has been assessed in terms of strengths, weaknesses, opportunities and threats.

## Results and discussion

### Mapping of existing data sources

Registers identified through both the literature review and the survey (82% response rate) have been classified by sectors of activity (table 2). This list does not represent an exhaustive list of all institutions collecting injury data in the EU. Not all of the reported injury/accident data collection systems are publicly available. Ideally, registers useful for consumer product safety and injury prevention would contain all of the following information: circumstances of injury (mechanism, intent, location, severity and time of injury, narrative); involved product (product category or more detailed description of product, eg, electrical tool, dispenser); brand of the product (detailed information on product identity and manufacturer) and information on the person injured (age, gender). However, only a very small number of identified registers were designed to intentionally collect such detailed information (eg, research project Susy Safe).

**Table 2:** Sample of data registers from different sectors of activity

Public healthcare-related registers The European Injury Database–Full Data Set Consumer Accident Monitoring (PL) Major Trauma Audit Methodology Odense University Hospital‘s Accident Analysis Group (DK) Enquête Permanente sur les Accidents de la vie Courante (FR) Observatorie MAVIE (My Life) (FR) Emergency department injury surveillance system (EL) Dutch Injury Surveillance System–Letsel Informatie Systeem (LIS) Dutch Hospital Discharge Registry (HDR) for hospitalised patients Trauma Audit and Research Network (TARN) (UK) The International Burn Injury Database (iBID) (UK) Global WHO Mortality Database European detailed mortality database (DMDB) (WHO) Clinical Practice Research Datalink (CPRD) (UK) European Health for All database (HFA DB) All Wales Injury Surveillance System (AWISS) National Information System on Health Infrastructure in the Domain of Primary Care (HR) National hospital care register (HILMO) (FI) TraumaRegister (DGU) (DE) Diagnoses Related Groups (DRG) (RO) Boulogne–Billancourt childhood injury database (FR) Styrian Injury Surveillance System (AT) Kind en Gezin, Regional project in Flanders (BE) Susy Safe Project–foreign body injury database (coord IT) Product-specific databases Database for ‘Dangerous Products in Germany’ Toy manufacturer: BRIO AB toy Industry (SE) Cosmetovigilance System (EU) RAPEX VARO system (FI) Road safety Community Road Accident Database-CARE (EU) STRADA—Swedish Traffic Accident Data Acquisition Polish Road Safety Observatory Database STATS19—personal injury road traffic accidents (UK) Central Database for in-depth analysis of road accidents (ZEDATU) (AT) German In-Depth Accident Study(GIDAS) Statistical database of traffic accidents—MALIS (ES) International Traffic Safety Data and Analysis Group—IRTAD Traffic accidents register—IPRVIS (LV) Firefighters and rescue services Swedish Civil Contingencies Agency (MSB)—IDA-Database (SE) Nordstat-fire incidents (DK, FI, IS, NO, SE) The Online Data Registration and Report System (ODIN) (DK) Incident Recording System (IRS) (UK)	Corpo Nazionale dei Vigili del fuoco database (IT) PRONTO (FI) Information system PÄVIS and supervision information system JÄIS (building fires) (EE) Norway fire incident reporting system—BRIS World Fire Statistics—Centre of Fire Statistics German Fire Protection Association (GFPA) Occupational safety registers European Statistics on Accidents at Work (ESAW) TOT—tutkinta (FI) Employer's Liability Insurance Association (DE) Danish Working Environment Authority Database for monitoring occupational accidents (ES) National Labour Inspectorate (NLI)–(PL) Information System for Occupational Injuries—ISA (SE) The Health and Occupation Research Network (THOR) (UK) Work accidents database (RO) Work accident database (HR) Sports National GAA (Gaelic Athletic Association) Injury Database Poison centres BfR poison information database (DE) Poison centres (sample of 8 across EU) Insurance companies National Health Insurance Fund-NHIF database ‘Sveidra’ (LT) The Claims and Underwriting Exchange (CUE) (UK) Insurance Europe association (EU) ANIA–Italian Insurers Association Croatian Insurance Bureau Polska Izba Ubezpieczeń/Polish Insurance Association Netherland Insurance Association German Association of Private Health Insurers Other registers ARIA Database of large scale incidents or accidents (FR) Energy-related Severe Accident Database (ENSAD) (CH) Hydrogen Incident and Accident Database (HIAD) (EU) Major Accident Reporting System (eMARS) (EU) Failure and Accidents Technical information System (FACTS) (global) Eurostat—injury and fatality statistics Consumer complaints registers National registers for complaints (Information received from the CNS survey) Online news EMM for retrieving information on unsafe products or accidents

European-centralised database Community Road Accident Database (CARE), Eurostat’s Statistics on Accidents at Work (ESAW) or cosmetovigilance systems are some of the examples of successful data collection practices across Europe which owe their success to both national efforts and well-defined and implemented legal framework that supports them. Many safety policy actions in the past have been driven by evidence from systematic data collection which in return triggered the development of safer products and services, which emphasise the relevance of systematic injury/accidents data collection. The above-mentioned systems (CARE, ESAW, cosmetovigilance) have been excluded from further analyses as they are managed under the umbrella of other policy frameworks and are already well established at the EU level. The Rapid Alert System (RAPEX) is an EU-wide rapid information exchange system on measures taken against consumer products (except for food, pharmaceutical and medical devices) that are identified to pose a risk. The system is not designed to provide information on the actual level of products’ safety in the EU. While notifying authorities are required to report on known incidents and accidents, in the majority of cases such information is not available. It has therefore not been further assessed in this study, due to the limited number of injury cases reported through RAPEX. The focus of this study was further narrowed down to existing data registers that have wide EU coverage, or from sectors whose activities are present in most of the EU Member States and/or could be deployed. By using this approach, the following data sources were analysed in more detail:

- European injury data base (IDB) (full data set (FDS)).
- Online news.
- FRS intervention reporting.
- PC records.
- Fatality statistics.
- Consumer complaints.
- Private economic operators (toy manufacturers, insurance companies).

### Potential of EU IDB-FDS for long-term support to consumer product safety

The EU IDB is a network which is running a database that holds standardised data on the external causes of injuries treated in emergency departments across EU.^27^ IDB was conceptualised in the 1980s for collecting data on home and leisure accidents (European Home and Leisure Accidents Surveillance System (EHLASS)). Its development was triggered by the success of the NEISS of the US CPSC in the 1970s. IDB’s initial purpose was primarily consumer protection, by the use of a well-developed data collection set called FDS to support consumer products safety policy.^28^ The classification system of the external causes of injuries, including the registration of objects involved, used in IDB is based on EHLASS (V.2000) and ICECI V.1.2 (WHO International Classification of External Causes of Injuries). It allows for a breakdown into a 1000 codes and 20 product groups (eg, furniture, child products, household appliances, items for personal use, sport equipment).^10^ Variables collected in the FDS are listed in table 3, showing this system’s capacity to code various ways of involvement of an object in an injury event (in bold). It is worth mentioning that the system does not provide information as to whether an injury has been caused by the product’s lack of safety, or its misuse, unless such information is provided in a narrative (as a free text) (table 3).

**Table 3:** The core variables of IDB-FDS*

Patient data	Specific data
Age and gender Place of residence Date of injury Date of treatment Nature of injury Part of body injured Assignment to further treatment	Injury mechanism Place of occurrence Activity when injured Types of sports, when sport **Object/agent triggering the injury** **Object agent causing the injury** **Other involvement of an agent** Details on road accidents Details on acts of violence Details on acts of self-harm **Narrative (120 characters**) National add-ons: eg, use of personal protective equipment, medication

Bold text highlights the capacity of the system to code different ways an object can be involved in an injury.

*Based on Kisser *et al*.^29^

FDS, full data set; IDB, injury data base.

In addition to the FDS that includes external causes of injuries and codification of objects involved in the incident, the IDB also contains the minimum data set (MDS) that is less costly to collect and contains data relevant for health and national health indicators. Data collection (FDS and MDS) is carried out by national authorities from hospitals/emergency departments who are members of the IDB network, which is coordinated by the European Association for Injury Prevention and Safety Promotion (EuroSafe). Once a year, the data are transferred from network members to a partner at Farr Institute (Swansea University, UK) for the quality check and, consequently, are fed into the database, which is hosted by the European Commission’s Directorate General for Health and Food Safety (SANTE).^27^ In 2016, according to the report published by EuroSafe, 10 Member States and Turkey uploaded their FDS into the IDB database.^29^ The number of participating members and hospitals varies slightly from year to year. Despite funding and operational challenges, the data from IDB-FDS are still used by some national authorities (primarily within the Ministries of Health). Over the years the IDB network has been cofunded under various EU funding schemes, such as the EU FP7 project INTEGRIS, JAMIE joint action and the BridgeHealth project, which ended in 2017, thus causing an uncertainty for its future operation.^30^ Currently, the use of data from IDB-FDS for product standardisation or research purposes is limited by strict data protection policy. Table 4 gives a list of the Strengths, Opportunities, Weaknesses and Threats (SWOT) which should be taken into consideration in the case of assessing the long-term potential of this network to provide data on product/object-related injuries, within its FDS.

**Table 4:** SWOT analyses of a selected source for long-term support to IT information platform on consumer product-related injuries and accidents

Data source	Strengths	Opportunities	Weaknesses	Threats
IDB-FDS (based on emergency departments data collection)	IDB network has a long-lasting expertise on injury data collection and analysis of external causes of injuries. This is an existing network that collects and analyses data on unintentional and intentional injuries from the emergency departments. The IDB methodology has been initially developed for the purpose of consumer product safety. The methodology and coding system for recording objects/substances involved in injury and capturing narrative is in place, well documented and publicly available.	The need for data on injuries by many stakeholders and the public. Implementing technical options at an affordable cost for improving the IDB-FDS data capture speed and the information coding (eg, voice recognition, automatic coding of injuries and external causes from narratives). Coordinated efforts with strong governance could bring the quality of data collection of all members of the IDB network on a comparable level. Data capture and upload frequency could be improved in the medium term. Data policy could be revised to allow easier access to microdata and narratives without compromising privacy.	Quality of data supplied to the IDB network by different member state (MS) can vary. Number of countries collecting IDB-FDS sometimes changes depending on the available funding in the MS. Highly restrictive policy applied by IDB network for the access to FDS data. Based on IDB report from 2016, 10 MS and Turkey collected FDS data. Frequency of data transfer to the central database (currently hosted by DG SANTE) occurs once a year. Brand and product names are not recorded. Follow-up studies are needed for more detailed information. Product 'involved' in injury does not mean 'unsafe' product. Case by case checks are required for determining the causality of an incident. Weaknesses of the system are also documented in EuroSafe and IDB publications.	Absence of a legal mandate that clearly stipulates responsibilities and ensures funding. Absence of a long-term sustainable governance mechanism (legally strong coordination point and management structures). Ending of funding under the BridgeHealth in 2017. Non-homogeneous level of interest by MS to provide further financial support. Increasing data protection concerns. Lack of clarity on how much funding is required to improve IDB to make it optimised and fit for purpose.
Firefighters intervention reporting registers	Firefighters and rescue services are in place in all Member States. All have developed reporting systems to their needs for fire prevention, allocation of resources, etc. Some authorities for consumer products safety already consult the firefighters records for identifying high-risk products (eg, FI).	Upgrading and standardising of intervention reporting systems could allow for more product-specific data collection. Scandinavian countries could be taken as examples of well-developed networks for sharing information on fire incidents (Nordstat). Have the potential for the retrieval of very specific product details on emerging unsafe products. Technical opportunities for interconnection and data centralisation.	In general, retrieving information on the unsafe product from firefighter's records is currently rather low. Registering details on products involved in accidents is often optional. No standardised data collection/reporting by firefighters at EU level. Relatively fragmented data source. No or poor standardisation of the terminology for the description of items or categories of the involved product. Caution in data interpretation: Not always clear relation between defective product and accident caused by negligence.	Possible lack of interest by Member States institutions to initiate collaboration on this matter, since consumer product safety is not among the primary objectives of fire intervention reporting. Low political interest/requirement for exchange of information.
Poison centres	Data collected at poison centres can contain information on high-risk products (mixtures, cleaning agents, etc). High amount of data available.	Recognition that harmonised poisoning case reporting is required and might be the logical next step after the introduction of the harmonised product notification template. From May 2017, manufacturers are obliged to report the composition of their liquid mixtures, as part of generation of UFI number.	Circumstances leading to the intoxication are typically not recorded (no time for that, treating the patient is priority). The product classification system is not compatible with other international classification systems (eg, ICECI). Accessibility of data is difficult due to confidentiality reasons. Data currently not collected in a harmonised way. Fragmented data source (many MS operate more than one poison centre, they may be organised on a regional level).	Low interest/requirement by national authorities to engage in exchange of information. Funding. Accessibility of data difficult due to confidentiality reasons.
Mortality statistics	Mortality statistics is one of the most complete, harmonised and already centralised data collection in Europe. Existing legal bases for mortality statistics. Large set of existing mortality data is available at EU level.	Death certificates have the option to record the underlying cause of death, such as injury. Electronic death registers could provide more opportunities for consumer product safety purposes in the future, if harmonised methodology for recording external causes of injuries is applied. Incorporation of ICECI system into ICD-11 could allow more comprehensive coding of external causes of injuries, if properly implemented. This data source could be combined with news media searching and death investigations to provide more details on involved products in fatal injuries.	Product-related causes of mortality are generally not registered in death certificates. Eurostat’s shortlist of external causes of morbidity and mortality has a limited range of non-disease-related codes (within ICD-9 and ICD-10) for registering causes of mortality, however these are not sufficient for selective product identification. Level of detail on the cause of death recorded in the certificate can vary. Data availability at EU level is not immediate (up to 24 months after the end of the reference year).	High data protection concerns. Legal obstacles in MS for carrying out inquiries into death circumstances. Availability of funding for death investigations.
Private economic operators (eg, toy manufacturer)	In-house registers of customer complaints contain specific information about safety issues with consumer products.	Manufacturers with higher market share could provide more information of interest to consumer safety policy work. Interest of private sector in using the available data on reported injuries caused by unsafe products. Dialogue with private economic operators needs to be initiated if their data sources are deemed of interest.	Data inaccessible for external users due to confidentiality principles. Highly fragmented source.	Low current interest of private sector to share data (sharing data is connected to vulnerability and potential loss of market). Very fragmented data.
Insurance companies	Hold some data on circumstances of serious injuries.	Insurance companies with wide coverage could be of interest as a potential data source if agreements are put in place for collaboration on collecting product-related information on accidents/injuries.	Insurance claims are generally not collected in a harmonised way neither at national level nor at European level. Purpose of insurance registers is estimating injury premiums prospectively. Product-related causes are generally not intentionally registered. Accessibility to data restricted due to data privacy and confidentiality reasons. Level of detail recorded in the claims is variable.	Lack of interest by insurance companies/insurance associations to share data. Insurance companies present very fragmented data source.
Consumer complaints	Existing EU regulation already stipulates the collection of consumer complaints by relevant national authorities. The channel of collecting this information has already been established in majority of MS (by email, phone, fax, allow sending videos).	Consumers engagement is encouraged in all EU MS. Technical opportunities allow for quick and easy reporting of injuries or potentially faulty products.	Collected information is submitted and collected following different formats across EU. Data analyses does not follow harmonised methodology (eg, product categorisation is different). The number of consumer product-related cases reported by consumers to responsible authorities varies greatly across EU. Reported cases need to be dealt with caution, since there is risk of misinterpretation/misinformation.	Low interest by MS in data sharing or harmonisation of data collection and analyses.
Online news search (EMM)	Automated system: update rate (24/7-hour service, variable refresh frequency up to 5 min). Can screen multiple languages. Allows for visual geographical data representation. Trend analyses. Can capture information on emerging risks or near misses from new products.	Already existing applications for online news retrieval (EMM exists from 2002). Systems for online news screening could be set up in relatively short term for monitoring the occurrence of fatal accidents or serious injuries related to consumer products. Relatively low-cost investment. Extending the monitoring from online media to social media might capture additional signals or trends (although content will need extensive verification).	Only the most sensationalised news, or most serious incidents make their way to the news. Having good online source coverage, such as regional and local news outlets is essential. What appears outside of the monitored scope will not be identified. Case by case manual check is required (to avoid risk of misinterpretation/misinformation). Additional investigations are required since product brand or circumstances may not always be available.	Disinformation campaigns (especially on social media). Lack of funding.

EMM, Europe Media Monitor; FDS, full data set; IDB, injury data base; SWOT, Strengths, Opportunities, Weaknesses and Threats; UFI, Unique Formula Identifier.

### Online media text mining using the EMM

Online news-based syndromic surveillance systems and the use of online review articles have already been developed for capturing information on public health threats and providing support to decision-makers.^31–33^ On this assumption, a pilot study has been carried out to automatically monitor online news articles for 3 months (29 September 2016 to 04 January 2017) with the aim to identify unsafe products, safety concerns or injury events. The EMM, an in-house developed tool, was used for monitoring selected online news outlets. The main operation principle of the EMM is based on setting a number of keywords, which when identified in an online news article classify and ‘capture’ the article as being of potential interest. News articles were classified as potentially being of interest only if at least one keyword from each four groups (see Methodology section) appeared in the online article. The ‘Toy’ product category was designed for the detection of safety concerns or injuries with toys, rather than generic items like ‘consumer products’, since preliminary analyses showed that monitoring of such a vague category as ‘all consumer products’ may result in many of incidents being undetected (due to large number of products for which suitable ‘Product’ keyword should be created). For the ‘Toy’ category, the EMM automatically collected approximately 250 articles in 3 months. Regular assessment and adjustment of the selected keywords finally resulted in a stable percentage of around 50% of relevant results which is likely the best result that was possible to obtain. In the same period, the percentage of false positives (news article is out of scope) dropped to a level of around 20% which is considered normal by the experienced users of EMM. The percentage of non-relevant results (contain all keywords, but not useful to product safety work) continuously decreased with each keyword list refinement, reaching around 30% in the last evaluations. Relevant articles predominantly contained information on product recalls, injury incidents, near-miss events or warnings on safety issues.

In conclusion, our study indicates that EMM has the potential to detect real-time news reporting about safety concerns or incidents related to the specific products, by focusing on a specific product category. If necessary, the system can be customised for other languages. Some limitations need to be taken into consideration:

▪ Despite the tool collects news articles automatically, results *must be verified and analysed manually* (to avoid misinterpretation or information misuse).

▪ Only the most serious accidents or sensationalised news find their way to the online news sources, therefore this source cannot be used for systematic or reference data collection.

▪ Manual update of new product search keywords may be necessary (eg, ‘fidget spinner’, ‘quadcopter,’).

SWOT analysis of this source to provide information on consumer product-related injuries/accidents is given in table 4.

### FRS intervention reporting in support of consumer product safety

FRS are often the first service to arrive at a serious accident, and their national or regional services maintain in-house registers for fire-related interventions. Main objectives of the fire intervention reporting are national statistics (eg, number of firefighters’ interventions per year, resources allocation, insurance claims’ predictions, firefighters safety). Based on the survey information and assessed data registers 39-48 (table 2), it has been observed that the data collection format for fire interventions is not standardised throughout Europe. Differences in intervention reporting can also be found between different regions of one Members State.

Intervention reports usually contain information on the cause of fire and cause of the ignition. If the cause of ignition was the failure of a machine or a device, more details can be provided. In addition, some systems allow ‘free-text’ option for describing the cause of fire, which could include the comprehensive information about the faulty/misused consumer product, however detection of unsafe consumer products is generally of low priority in FRS intervention reporting and is carried out on a voluntary basis (optional) by the responsible fire officer. Some advanced and centralised systems for data collection have been reported to us from the survey respondents. One such system is PRONTO (Finland), a database that collects data from 22 rescue departments, contains detailed accident narratives and may contain information on the products involved, so that the Finish national authority for consumer safety and researchers can already make use of these data. Other interesting examples are Swedish IDA (//ida.msb.se), Danish Online Data Registration and Reporting System (ODIN), Incident Recording System in UK, BRIS system in Norway, to name just a few, which all to a different extent collect information on the product/machine/activity that caused a fire. In some cases, establishing the cause of a fire is determined by the police investigation, which makes completing FRS intervention reports more complex. Our interviews indicated that there is a willingness by certain firefighter associations to promote moving towards more harmonised data collection in the future, which could allow the harnessing of useful information on faulty or high-risk products by many other stakeholders. Unlike some other statistics collected under national authorities, there are no guidelines from Eurostat on the collection of fire incidents data.

The availability and accessibility of the information from FRS registers also varies between countries. Some countries provide part of their registers to the public through online collaborative portals such as Nordstat (www.nordstat.net).

The relevance of FRS intervention reporting for product safety purposes could be increased by harmonising collected information, with a focus on identification of faulty/misused products and circumstances that have caused fire incidents (table 4). Further exploration of the potential and the cost of this sector to provide information on high-risk products involved in fire incidents could be undertaken by means of a pilot project.

### Potential of PC registers across EU to provide data on unsafe consumer products

PC play an important protective role in the use of products containing chemical mixtures. In case of exposure to hazardous chemicals, they provide medical advice to general consumers and physicians. It has been estimated that on average these services at the EU level receive and treat 600 000 calls per year, which is almost 1700 calls per day, mostly related to child exposure. The number of fatalities related to chemical exposure is considered to be over 400 per year. Many Member States operate more than one PC, and in some countries they are organised on a regional level.^34^

Poisoning incidents are usually documented following a qualitative methodology based on internally standardised documentation of cases. Which data should be collected as a minimum standard has frequently been discussed by members of the European Association of Poison Centres and Clinical Toxicologists, but no formal agreement at the EU level has been reached so far. All PCs typically collect a basic set of information that are essential to do the risk assessment and provide the caller, as soon as possible, with the relevant treatment information. The collected data include some information on the substance or product category involved (eg, home detergents and cleaning products, cosmetics) and its toxicological ingredients/quantities.

Companies placing mixtures on the market have the legal obligation, according to Article 45 of the Regulation (EC) No 1272/2008 (CLP Regulation) to inform appointed bodies (Governmental Authorities or Poison Centres) about the composition and the toxicological properties of mixtures classified as hazardous. On 22 March 2017, the Commission adopted the Regulation (EU) No 2017/542, which amends the CLP Regulation by adding an Annex harmonising information related to emergency health response. The Regulation will require producers and importers of chemical mixtures (such as detergents, paints and household chemicals) to provide information on the product composition, through a Unique Formula Identifier on the label of the product, thus allowing a precise and rapid identification of its specific chemical formulation by an emergency health response operator (eg, PC). Regulation (EU) No 2017/542 is going to fully apply from the 1 January 2020 for chemical mixtures for consumer use.

At present, PC registers may only be used to meet medical demand by formulating curative measures and were requested by the Member State to undertake statistical analysis to identify where improvements to risk management measures may be needed. Confidentiality issues concerning individually registered cases likely concern personal data regulated in both national and European legislation. Accessibility could be explored on condition of anonymisation of the personal information. Considerations on the strengths and weaknesses of this data source to support product safety work are presented in table 4.

In conclusion, data collected at PC contain valuable information on chemical mixtures or liquid consumer products. However, circumstances leading to the intoxication are typically not recorded in a harmonised way and data accessibility is difficult due to confidentiality reasons. Our analyses indicate that direct exploitation of data from PC is not feasible in the short term, without prior attempt for harmonisation among existing data collection methodologies and clarification about the conditions of data accessibility.

### Mortality statistics in support of consumer product safety

Mortality statistics is one of the most complete and accurate epidemiological data collection practices in Europe. In all EU countries, the medical certification of death was made mandatory according to the Commission Regulation (EU) No 328/2011 on COD statistics. Member States are required to provide the data specified in this Regulation to the Commission (Eurostat) within 24 months after the end of the reference year. Coding of the COD is performed by the use of a harmonised methodology and coding system (ICD-9 and ICD-10). Most countries in the world and the EU use the WHO international standard form for describing the COD, which allows for the registration of the underlying event or injury that initiated the chain of events causing the fatal outcome (part I of the WHO COD form). The extent of detail on the non-medical circumstances of the injury event (external cause of morbidity and mortality), in the COD, is usually poor and does not allow for simple identification of a potentially involved product or a product category from this type of data source. Trends and inconsistencies in filling the mortality certificates have already been reported and efforts to increase the accuracy of the information recording are ongoing.^35–37^

For example, based on the Eurostat shortlist of external causes of morbidity and mortality, ICD-10-coded information such as the one listed below could be identified from the death certificates:

W20 ‘Struck by thrown, projected or falling object’,

W21 ‘Striking against or struck by sports equipment’

W36 ‘Explosion or rupture of a gas cylinder’

W87 ‘Exposure to unspecified electric current’, etc

The above listed examples show that the information on the cause of the mortality (by the use of ICD-10) that can currently be recovered from the death certificates is not specific enough to support consumer product safety work. In rare cases, some indication on limited range of product categories (eg, sports equipment) may be retrieved (if the form is properly filled), but no specific details on the product can be recovered. Additional death investigation studies would be necessary for obtaining more details on the circumstances or products involved in the fatal accidents, such as using medical records, exploring online news sources or contacting the family of the deceased. Similar work is being carried out by the US CPSC.^38^ In Europe, the use of information from the death certificates for research or death investigations is still heavily regulated by national laws on data privacy, therefore the use of this potential data source is not feasible in the short term.^39^ A SWOT analyses of this potential data source is given in table 4.

Potentially, Eurostat’s shortlist of external causes of morbidity and mortality could be amended to satisfy desired granularity or focus on specific type of injury that is involving objects/consumer products. This would require collaboration with a wide range of stakeholders from the Member States. The development of electronic death registration systems may provide more opportunities for data usage from mortality statistics in the future. In addition, the use of the new coding system ICD-11, that is in preparation under the umbrella of WHO, may allow improved coding of external causes of injuries (by linking the ICECI coding system with the ICD module for external causes) and therefore higher disaggregation of collected data.^9^

### Consumer complaints in support of consumer product safety

According to the GPSD (2001/95/EC), Article 9,^2^ Member States’ authorities are required to provide opportunities for submitting consumer’s complaints on product safety. The survey with CSN was focused on finding out how product safety complaints from consumers are collected and analysed. Eighteen Member States CSN representatives replied to the survey (56% response). The responses indicated that all institutions allow for electronic submission of complaints in various forms (downloadable forms, online form, photos, videos), and also by phone or fax. It has been observed that responsible jurisdictions have different ways of analysing data, categorising injuries and products. Some CSN members reported the use of classifications by in-house coding systems for products which contain elements of RAPEX and international standardisation codes or reported using LOTUS system for registering emails and performing prioritisations based on the severity risk. The number of product-related consumer complaints reported to the responsible authority varied considerably per country. The survey indicated different views on the subject of sharing data on complaints among the members of the CSN. A third of the survey respondents expressed that they would share their data on complaints on unsafe products for the purpose of research or policymaking. The observed strengths and weaknesses of the existing system of consumer complaints is provided in table 4.

An interesting IT system has been reported by a Slovakian member of CSN on teaching citizens how to be proactive in product safety and market surveillance: ECHO system–consumer information on dangerous products (available at soi.sk). An information system called ECHO was launched in 2007 with the objective to gather information on accidents caused by the use of any non-food products in the household, in leisure and in sport activities. The system has an educational element in itself to promote more active communication with the consumers and the market surveillance body.

Results of the survey suggest that national authorities should continue to operate and innovate their systems for collecting consumer safety complaints. The quality of product-related injury/accident data could be increased by harmonising the procedures/methodologies for receiving safety complaints from consumers and then potentially forwarding details of the incident and product concerned to an EU-wide integration platform. The obstacles for anonymised data sharing should be explored.

### Can the private sector contribute to data on product safety?

#### Case study with toy manufacturers

With the assistance of the association Toy Industries of Europe, one large toy manufacturer provided information on handling data on product safety customer complaints. The toy company provided information on their in-house data collection system in which they store data such as claims, complaints, the batch code of the involved product, the injuries caused by their products, the circumstances which lead to the injury and the customer contact details. The data entry input mask includes free-text boxes where the occurred injury can be described in detail. The most common complain reason can be selected from a pull-down menu. Most information stored in this system is provided by the customers directly, the reseller or company agents. Many of the larger toy manufacturers hold in-house registers of customer complaints and on faulty products, however the data have limited access.

#### Insurance companies

Insurance companies were thought to be an interesting potential source of information on product-related causes of injuries and/or death. Types of insurances which were addressed for this purpose were umbrella insurance associations, personal accident insurances, medical and health insurances. The information recovered by survey from the representatives of associations/companies from sources 62–69 (table 2) suggests that the use of the data from insurance companies could be quite challenging.

Details on the circumstance of accidents, including some detail on possibly involved consumer products, may be held at the private insurer level, however all companies will have their own methods of data compilation and statistical data analyses. Insurers would not intentionally collect information about a faulty product. Information on the external cause of accidents/involved products, if any, would not be shared at the higher level such as with roof insurance associations. Other than that, data protection concerns and high fragmentation of this sector would provide major obstacles for the direct usage of data in the short term. Other observations are given in table 4.

The interest of the private sector to collaborate and share information was low in the course of this project. The concerns about sharing data/information were mainly associated with the potential of data misinterpretation and/or unfair stigmatisation of products/companies. On the other hand, private sector representatives showed interest in using the available data on injuries caused by unsafe products. There are currently ongoing discussions on data sharing in a business-to-government context between the Commission services, stakeholders and the involvement of newly appointed Experts Group on Business-to-Government Data Sharing.^40 41^

### Data needs

When developing a new data infrastructure, preliminary analyses of the stakeholders data needs are necessary in order to understand if target objectives by different stakeholders can be met (eg, increasing safety of a product, injury prevention, risk reduction, monitoring of legislation implementation, standardisation work).

Detailed analyses of methodological approaches and pitfalls to consider before setting up systematic collection of statistics for the safety of services have been previously published in a report commissioned by the European Commission’s DG SANCO.^42^ Based on this report, some considerations on the data/information needs by various stakeholders have been indicated in table 5. Defining the minimum, but sufficient number of parameters and quality of data, that are fit for purpose, is essential for rationalising data collection efforts. This is equally valid for developing new data infrastructure or deployment of the existing databases.

**Table 5:** Considerations on stakeholders data need*

Stakeholders	Purpose of data usage	Information needed	Coverage of data set	Frequency of update
EU institutions	Legislation and policy development Establishing need for action Setting work programme Allocating resources Priorities setting Monitoring and impact analyses Assessment of burden on public health and individual consumer risk Drafting mandates for standards Injury prevention strategies	Quantitative and qualitative data on incidents rates, the role of human intent, mechanism, identified unsafe products, consumer risks Number of fatalities and severe injuries Emerging dangerous products	EU coverage Vulnerable populations Specific sectors (toys, cosmetics, DIY tools, etc)	More frequent data sets and annual trends (real-time monitoring from online news sources or social media). 3, 5, 10 years for policy update assessment
MS and regional consumer product safety jurisdictions	Enforcement work Work programme setting Resources allocation Establishing national and regional safety/injury prevention actions, education and awareness programmes, etc Progress monitoring and impact analyses	Quantitative and qualitative data on number of injuries, the role of human intent, mechanism, unsafe products, consumer risks Focus on fatal and severe injuries, emerging dangerous products	Regional or national coverage	More frequent data sets for emerging safety issues or fatalities (preferably real time) Usually annual update for trends assessment
Standardisation bodies	Drafting standards/codes of practice	Qualitative and quantitative data on injuries (from scientific literature, or simple case listings with accidents description) Fatal accidents investigations	Regional, national, international	Not very frequent, when required by consumer associations, national or EU jurisdictions, or due to major revision of standards
Consumer protection associations	Awareness-rising efforts Identification or collecting evidence for changes in the regulation or new standards	Quantitative and qualitative data on complaints, accidents, safety testing	EU/national/regional	When necessary or annual
Insurance companies	Assessment of risks and premiums Resources allocation	Accurate data on the risks of injury, injury studies In-house risks assessment	Risk data per sector (eg, leisure sport activities) Regional or across EU Vulnerable groups	As needed, premiums adjusted by trend analyses of claims
Firefighters and rescue services	Allocation of resources and safety of firefighters Developing strategies for fire reduction	Intervention reporting data Cause of fire	Regional, national	Annual for work programmes and resources allocation
Poison centres	Allocation of resources Assessing risks of poisoning Identification of hazards	Quantitative data on number of cases treated Poisoning circumstances	Regional, national	Annual work programme assessment

*Example is adaptation based on Hayward.^42^

### A platform for remote data access

**Figure 1:**
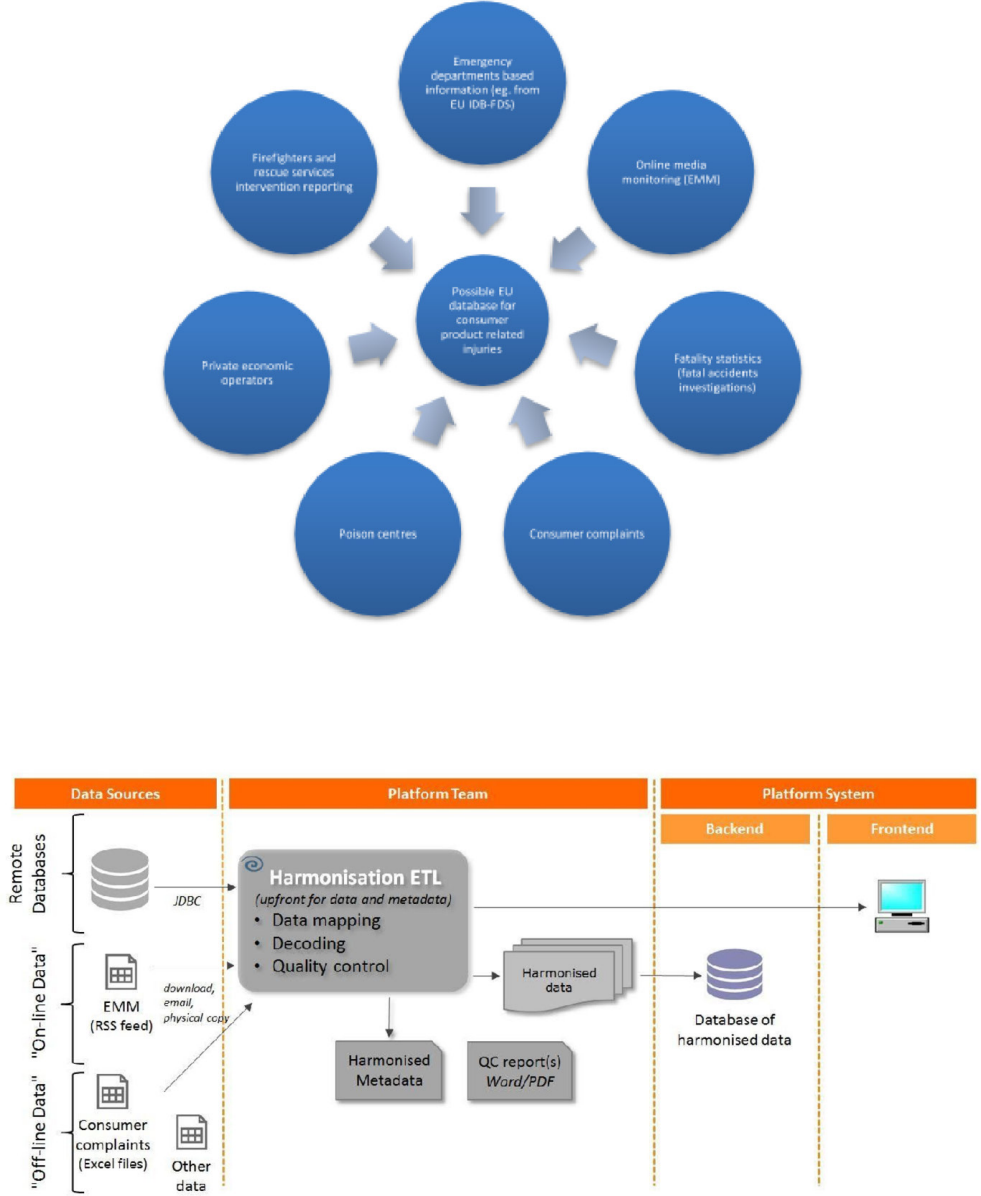
Proposed data flow diagram and options for data access by remote linking of potential data sources onto a common information platform

Based on the priority data sources that have been identified and assessed here as being of potential interest to the consumer product safety work, we propose to put in place an IT platform for remote access of data in a modular-based fashion. A successful example of a distributed remote data access via IT platform, in the area of environmental chemical occurrence data, is the European Commission’s Information Platform for Chemical Monitoring (IPCHEM). IPCHEM has been recently recognised by the Parliament as an initiative reducing the knowledge gap on the environmental data, related to the implementation of the 7th Environment Action Programme.^43^ IPCHEM allows remote access to data from various providers such as European Agencies, Members State authorities and research institutions who committed to support data sharing policy, in the spirit of collaborative initiative called Shared Environmental Information System (SEIS).^44^ Similar to IPCHEM, the possibility of modular interlinking of structured, semi-structured and unstructured data, which can be accessed remotely, while taking advantage of IT technologies, could be applied in the field of consumer product safety. This would imply working with data coming from various data sources, such as fatality statistics and consumer complaints, complemented with relevant online news articles/customer reviews (eg, EMM-retrieved information) for detecting emerging risks and trends. In the long term, other sources such as harmonised fire and rescue service records/PC or information supplied from the private sector could also be integrated, if required. Basic data flow and options for remote data access or the use of online and offline data sources delivered by data providers are visualised in figure 1.

Furthermore, novel IT paradigms of interlinking different data sources such as the *Semantic Web* using a *Linked Data approach* could be explored.^45^ However, it should be stressed that such option would require major collaborative efforts (technical, organisational and in terms of governance) across participating data infrastructures and information systems.

In our estimate, the functional use of data from assessed sources, apart from EMM, cannot be achieved in the short term (ie, less than 5 years). Due to limited time and resources available for this project, it was not possible to perform a cost-effectiveness analysis of the various options. However, such analysis is necessary to be able to conclude on the feasibility of the proposed options.

## Conclusion

This study aimed to assess the potential for increasing the availability of data and information on consumer product-related injuries or accidents, by the deployment of already existing injury data and by exploiting ‘alternative’ data registers from different sectors of activity, online news monitoring and information interlinking onto a common information platform.

The following data sources have been identified to be of potential interest: injury data collected from hospitals’ emergency departments held by EU IDB within its FDS, death certificates, firefighters’ records, PC registers, consumer complaints, online news sources, insurance associations and private economic sectors. These data sources present pros and cons in terms of details on the involved product and the circumstances surrounding injuries, regional fragmentation, coverage, methodologies, terminology, willingness for data sharing, risk of data misinterpretation or misuse (eg, fake news, information manipulation).

At the European level, a long-established IDB network is already collecting and storing cross national data on injuries (intentional and unintentional), collected mostly from emergency departments. The FDS of the IDB could provide systematic information on external causes of injuries, if its weaknesses are tackled. The absence of a legal mandate for a long-term systematic collection of IDBs’ FDS data, along with the lack of sustainable funding and the increasing data protection concerns, may be regarded as the main reasons currently hampering the full exploitation of the capacity of the IDB network to serve consumer product safety purposes. However, cost–benefit analyses of the necessary optimisation effort needed to increase its capacity to support consumer safety work should still be carried out.

The other potential data sources identified in this study are the intervention records of the firefighters or PC. As these data sources are tailored for different purposes that are not primarily focused on recording the potential involvement of consumer products in incidents, they are not ready to be used as such in the short term. These data collections should first be standardised, harmonised and brought up to the desired level of quality. Such a task, to be successful, would require substantial coordinated efforts over an extended time frame.

To respond to a demand for real-time information on consumer product-related injuries or accidents, a pilot study with the use of the EMM for online news monitoring has been carried out. EMM could be useful for extracting information on emerging consumer product safety concerns reported in the local online news sources or social media in any of the EU languages and thus support market surveillance and the decision-making community or inform consumers. Online news articles could be used to complement investigations into COD in terms of consumer product involvement in fatal accidents. In addition, harnessing information from consumer complaints submitted to the national authorities could also provide useful information on emerging product safety concerns. However, caution is needed when using online sources or consumer complaints to avoid data misinterpretation or unfair stigmatisation of a product manufacturer.

With regards to the private sector, setting up a business to government data-sharing landscape that is mutually beneficial could motivate stakeholders from this sector to share their data on consumer product-related injuries or accidents (eg, product manufacturers, insurance companies).

IT data-interlinking options, such as remote data access, can provide opportunities for enhancing information availability, while respecting data protection issues (eg, data anonymisation) and without compromising data ownership. The selection of the most appropriate data sets should, in principle, depend on the identified data needs by primary stakeholders, by taking into account considerations such as the impact on risk reduction, product safety or accident prevention. A more detailed analysis needs to be carried out to conclude on the feasibility, cost–benefit ratio and impact of the proposed options.

Consumer product safety issues are highly cross-sectoral and the objective of successfully increasing the availability of relevant data can only be achieved if the collaboration and active involvement of all stakeholders concerned can be ensured (national authorities for consumer safety and injury prevention, European Commission services, consumer associations, manufacturers, members of public, etc).
